# The effects of 12 weeks of functional strength training on muscle strength, volume and activity upon exposure to elevated G_z_ forces in high-performance aircraft personnel

**DOI:** 10.1186/s40779-021-00305-8

**Published:** 2021-02-23

**Authors:** Monika Rausch, Frank Weber, Sven Kühn, Carla Ledderhos, Christoph Zinner, Billy Sperlich

**Affiliations:** 1 Multinational Medical Coordination Centre/European Medical Command, Andernacher Straße 100, 56070 Koblenz, Germany; 2 German Air Force Center of Aerospace Medicine, 82256 Fuerstenfeldbruck, Germany; 3 Department of Radiology, Bundeswehr Central Hospital, 56072 Koblenz, Germany; 4 Department of Sport, University of Applied Sciences for Police and Administration of Hesse, 65199 Wiesbaden, Germany; 5 Integrative & Experimental Exercise Science & Training, Institute of Sport Science, University of Wuerzburg, 97082 Wuerzburg, Germany

**Keywords:** Fighter pilots, Long-arm centrifuge, Muscular strain, Strength training, Neck muscle, High-performance aircraft, G_z_ forces

## Abstract

**Background:**

Technological advancements in modern military and acrobatic jet planes have resulted in extraordinary psychophysiological loads being exerted upon flying personnel, including inducing neck and back pain. The purpose of this study was to examine the effects of 12 weeks of functional strength training on 1) the volume and strength of the neck and shoulder muscles and 2) muscular activity upon exposure to helmets of different masses and elevated G_z_ forces in a long-arm centrifuge in high-performance aircraft personnel.

**Methods:**

Eighteen participants underwent 12 weeks of functional strength training (*n* = 12) or the control protocol (*n* = 6) without additional strength training. Pre- and post-intervention tests included evaluations of isometric strength of the head extensor muscles, flexion, and lateral flexion and rotation, as well as magnetic resonance imaging (MRI) to measure the volume of the *m. sternocleidomastoideus*, *m. trapezius*, and deep neck muscles. Furthermore, during a long-arm centrifuge (+ 1.4 and + 3 G_z_) protocol, the muscular activity levels of the *m. sternocleidomastoideus*, *m. trapezius* and *m. erector spinae* muscles were assessed without a flight helmet, with a helmet, and with a helmet and night vision goggles. Each participant’s perception of muscular strain was noted immediately after the long-arm centrifuge protocol.

**Results:**

The maximal isometric strength in all exercises and muscle volumes increased in the training group but not the control group (*P* < 0.05). Relative muscle activity (%MVC) with a helmet decreased after the intervention in the training but not the control group (*P* = 0.01). Relative muscle activity while wearing a helmet and night vision goggles was higher after intervention in the control group than in the training group (*P* < 0.01). The perceived muscular strain of the neck muscles induced by the long-arm centrifuge did not differ between the groups.

**Conclusion:**

Twelve weeks of functional strength training improves the maximal isometric strength and volume of neck and shoulder muscles and leads to lower relative muscle activation upon exposure to elevated G_z_ forces in a long-arm centrifuge.

**Supplementary Information:**

The online version contains supplementary material available at 10.1186/s40779-021-00305-8.

## Background

Technological advancements in modern military and acrobatic jet planes have reached a level that has resulted in the exertion of extraordinary psychophysiological loads upon flying personnel. Pilots of modern fighter or acrobatic aircraft may tolerate angular forces of up to + 9 G_z_ during cornering at high velocity, resulting in a perceived mass 9 times heavier than the actual mass (e.g., a 70 kg body mass being equivalent to ~ 630 kg). Two major processes influence a pilot’s performance at high + G_z_: 1) the shifting of blood from the brain towards the lower extremities, leaving the pilot subject to loss of consciousness and control of the aircraft; and 2) a significantly increased risk of musculoskeletal injury [1–4].

The most prominent risk factors for spine injuries among high-performance aircraft personnel are 1) cumulative exposure to elevated + G_z_ during flights; 2) unpredictable acceleration variation; and 3) the adoption of nonneutral positions of the cervical spine through head rotation during high + G_z_ [5]. The incidence of spine injury increases due to more flight hours, lack of physical fitness, increased pilot age and, above all, the mass of modern helmets and night vision goggles [3–7]. Most complaints are not caused by pathological processes but emerge as muscular, nonspecific neck and back pain [8, 9].

Many minor injuries of the musculature or tissue occur at relatively low acceleration forces (approximately + 4 G_z_), especially when the acceleration changes unexpectedly [8]. Since personnel in high-performance aircraft cannot change their posture due to the design of the cockpit, the human factor of the human-machine interaction warrants special attention and is subject to optimization. A large body of medical aviation studies dealing with spinal issues arising from high-performance aircraft exposure has concluded that future research should focus on the mass of the helmet and special training interventions such as strength training of the back and neck muscles to counteract the muscular stress on the cervical spine during high-performance flights [10]. Based on previous findings [1, 11], it seems reasonable to assume that augmentation of the size and strength of the muscles stabilizing the spine could protect against G-induced neck and back strain.

At present, no evidence-based exercise program exists to specifically improve the strength of the muscles around the spine to reduce physiological strain and perceived stress in flight-specific hypergravity conditions. Since physiological experiments in high-performance aircraft are expensive and may require cumbersome measurement devices, most experiments on high-performance aircraft personnel are carried out in centrifuges capable of inducing elevated G-forces and simulating flight-like settings. Therefore, this study aimed to examine the effects of a 12-week functional whole-body strength exercise routine on the 1) volume and strength of the neck and shoulder muscles and 2) muscular activity and muscle strain upon exposure to flight-specific hypergravity conditions induced by a centrifuge in high-performance aircraft personnel.

## Methods

### Participants

Eighteen participants, i.e., 3 active jet pilots and 15 novice pilots with no or marginal experience with exposure to G-forces, volunteered to take part in this study. Twelve participants were assigned to the training group (one female participant; age 31 ± 11 years; body mass index 22.4 ± 1.9 kg/m^2^), and 6 individuals were assigned to the control group (one female participant; age 30 ± 8 years; body mass index 24.7 ± 2.4 kg/m^2^). This study was performed in accordance with the Declaration of Helsinki and approved by the Ethical Committee of the Sports Institute of the University of Wuerzburg.

### Study design

During the first visit, all participants underwent a special medical examination at the aviation facility for aeromedical fitness to confirm that they were qualified for hypergravity testing. One day later and after positive aeromedical assessment, the volume and strength of the neck and shoulder muscles were determined by magnetic resonance imaging (MRI) and a specialized strength apparatus. Within the next week, all experiments at a high + G_z_ were performed in a long-arm centrifuge. After the 12-week intervention, each participant repeated all testing procedures except the aeromedical examination, as the results of this examination are valid for one year.

### MRI

MRI was performed on a 3 T scanner (Magnetom Prisma, Siemens, Erlangen, Germany) employing a 64-channel head-neck coil. Careful attention was given to ensure that the head, shoulder and neck were placed in identical positions during pre- and post-intervention measurements by measuring the distance between the chin and the jugular vein. All volume measurements were taken using a three-dimensional volumetric isotropic T_1_-weighted vibe DIXON sequence with a voxel size of 1 mm^3^. An experienced researcher performed all measurements using three-dimensional Slicer Software (v. 4.8).

A semiautomatic threshold technique was used for volume extraction. First, a threshold value range of the signal intensity for muscle parenchyma was defined to ensure that only voxels representing muscle were selected when segmenting them from surrounding tissues. Then, the muscle outline was drawn slice by slice in the axial direction. The 0 % distance factor between the slices allowed a full volume without any gaps. Although numerous studies simply measured the cross-section in one or a few slices, we aimed to obtain high accuracy by a gapless determination of the total volume since previous reports [12] have shown that a complete volumetric assessment is more accurate than partial measurements. Afterwards, the generated outline was checked and corrected in the coronal and sagittal planes, and an integrated algorithm smoothed the contours to simulate the surface of the muscle. To determine the volume of each muscle, all voxels contained within the three-dimensional form were automatically summarized.

### Maximum isometric strength

Isometric maximum strength forces were obtained for all main directions of movement of the cervical spine (extension, flexion, right and left lateral flexion, and left and right rotation) employing a specialized strength training apparatus (DiagnosMed 2000, Schnell Trainingsgeräte GmbH, Gachenbach, Germany) before and after the 12-week intervention.

As in another study [13], for isometric strength testing, an experienced sports scientist required the participants to exert maximum force against the dynamometer lever, which was adjusted according to the individual’s anatomy, as quickly as possible for 3–4 s. Verbal encouragement was given to ensure maximal effort. Three trials were performed for each movement, with a 1-min rest between trials. The highest maximal voluntary contraction force was used for statistical analysis.

### Long-arm centrifuge protocol and electromyographic assessment

**Fig. 1:**
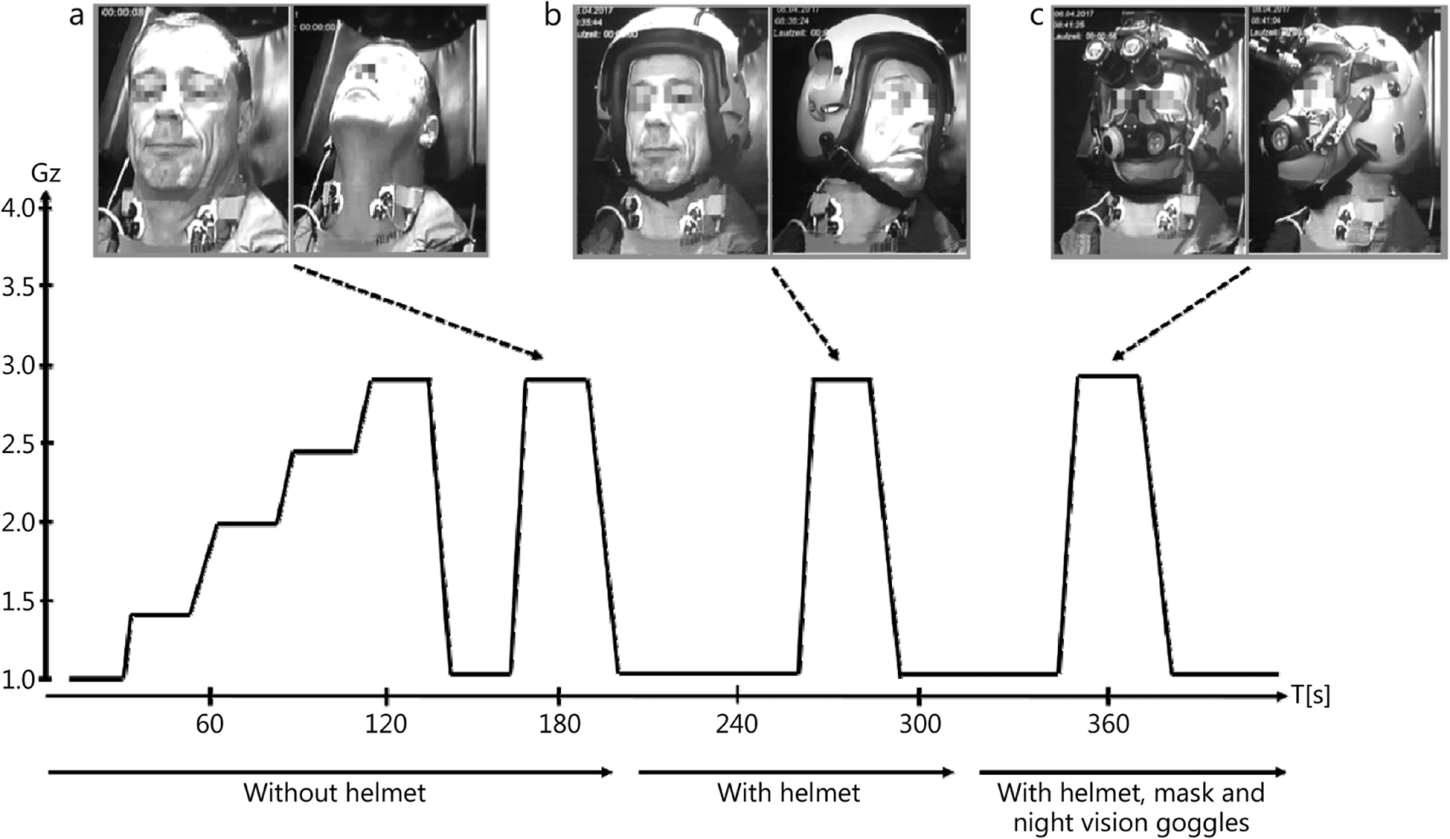
Illustration of the different head movements performed during the long-arm centrifuge protocol. **a**. Head movements from the neutral position upwards at + 3 G_z_ without a helmet. **b**. Head movements from the neutral position to the left at + 3 G_z_ with a helmet. **c**. Head movements from the neutral position to the right at + 3 G_z_ with a helmet, mask and night vision goggles

In this investigation, all participants performed three long-arm centrifuge protocols with G_z_ forces from + 1.4 G_z_ to + 3 G_z_ under three different conditions (Fig. 1): without a helmet (= head mass), with a helmet (= head mass + 1.4 kg), and with a helmet, visor, mask and night vision goggles (= head mass + 2.3 kg).

The helmets were the type used in Tornado aircraft and included visor and night vision goggles (Model: HGU-55 IG), and they were provided by Gentex (IEA Mil-Optics GmbH). The centrifuge had a long (9.5 m) rotating arm with a gondola and had actively powered cabin pitch and roll. In this research, no flight simulation was used, but a standardized profile was chosen, which began with a familiarization profile to obtain the subjects used to acceleration. Measurements were then carried out at a linear acceleration and an onset rate of + 0.1 G_z_/s up to + 3 G_z_. During the three long-arm centrifuge protocols, the participants were monitored by an experienced centrifuge physician and received instructions from a sports scientist at the controller’s desk via a speech connection to the centrifuge. They were instructed to keep their heads in a neutral position from + 1.4 G_z_ to + 3 G_z_ (head straight, looking forward). When + 3 G_z_ was reached, they were instructed to turn their heads upwards, to the left and to the right.

A limit of + 3 G_z_ was chosen, and the subjective G-tolerance averages were between + 3.5 and + 4 G_z_ [14]. Since most of the participants were beginners, no anti-G suits and no positive pressure breathing were used to avoid confounding effects and to strain the subjects only as much as necessary without anti-G protective devices [14]. Head rotations were also performed, for which the risk of injury increases at higher accelerations.

Figure 1 illustrates the three different settings used during the long-arm centrifuge protocol at + 3 G_z_: head in the neutral position (without a helmet), head movement to the right (with a helmet), and head movement to the left (with a helmet and night vision goggles).

During the long-arm centrifuge protocol, the electromyographic activities of the *m. sternocleidomastoideus* muscle and the descending part of the *m. trapezius* and *m. erector spinae* muscles were measured. For inter- and intraindividual comparisons, the maximum voluntary contraction (MVC) normalization method was applied for each muscle, and the same electrodes and placements were used during pre- and post-intervention testing. Bipolar electrodes precoated with gel were placed on the bellies of these muscles in alignment with the underlying muscle fibers and in accordance with international standards [15]. MVC was determined by head rotations against a resistance belt positioned on the head for the sternocleidomastoid muscles, shoulder raises against a resistance belt for the upper trapezius muscles, and cervical extension against a wall for the cervical erector spinae muscles.

A wireless 16-channel device was used to assess electromyographic activity during the experiment (Telemyo-DTS, Noraxon Inc., Scottsdale, AZ, USA). Based on the recommended scientific standards for surface electromyographic measurements [16, 17], the device was adjusted to have a bandpass filter of 10–500 Hz and a sampling rate of 1500 Hz. Silver/silver chloride-based disposable electrodes (Noraxon dual electrodes) with an interelectrode distance of 175 mm were used. MyoResearch 3.0 analysis software was used to record and process the raw data, specifically the “MyoMuscle” EMG module (Noraxon Inc., Scottsdale, AZ, USA).

### Perception of muscle strain

The question “How strained do your neck, shoulder and upper back muscles feel during the centrifuge runs?” was asked to obtain self-reports of perceived muscle strain after each long-arm centrifuge protocol from each participant. The participants responded using a five-point anchored scale: 1) highly stressed, 2) stressed, 3) less stressed, 4) no stress, and 5) no statement.

### Twelve-week functional strength training program

The functional strength training program, which consisted of multiple whole-body strength routines emphasizing the neck, abdominal and shoulder muscles, is summarized in Supplemental Table 1.

During the 12-week training program, all participants performed three different workouts three times per week (Supplemental Table 1). Each workout lasted 60 min and was divided into a 10-min warm-up followed by 40 min of strength training of 12 to 15 repetitions (until muscle fatigue was reached) and a 10-min core stability phase. As we intended for the strength training program to be able to be completed anywhere (i.e., at home or on base), all necessary equipment had to be able to be stored in a normal duffel bag. Thus, no machine-based strength exercises were included in this experiment.

Each participant received an individual introduction for every training session and a detailed training plan with a precise video-based description of each movement. The exercises included neck flexion, extension, and rotation exercises. The intensity was controlled by the equipment, which included a sandbag, medicine balls, bands, and small weights. All sessions were documented in a training diary. The participants of the control group were instructed to adhere to their usual activities of daily living.

### Statistical analysis

The maximum isometric strength test, volume and strain perception data are presented as the mean *±* SD. A Shapiro-Wilk test was employed to assess data normality, and no further transformation was necessary. To assess differences between or within the two groups between the pre- to post-intervention test results, a, independent or paired samples Student’s *t*-test was employed using SPSS 22.0 analysis software. The effect sizes (*d*) were calculated using the differences of the mean values, and the standard deviations and were interpreted according to Cohen [18].

Due to the multivariate nature of EMG data, a linear mixed effect model (LME) was applied as described elsewhere [19, 20]. LME has the advantage of being less vulnerable to missing data points and is well suited for repeated measurements. In the present model, the three muscles were the dependent variables. We used acceleration, helmet, group, test time (pre/post) and head movement as explanatory variables and fixed effects, while the participants were used as the random effects. The volume of the measured muscles and the corresponding maximum strength test results were defined as response variables. Bonferroni correction for multiple comparisons was used, and the LME models were optimized according to the Akaike information criterion. A model was accepted if the residuals were normally distributed; otherwise, the model was rejected, and the data were evaluated by applying the parametric or nonparametric test procedures mentioned above. Significance was set as *P* ≤ 0.05. The effect sizes were calculated using *F*-values [19].

## Results

Participants in the training group performed 27–31 sessions, i.e., 80% of all possible sessions, during the intervention. All mean values, % changes, *P*-values and *d* values for the various volume and strength variables for both groups before and after the 12-week intervention are summarized in Table 1.

**Table 1:** All variables of the training and control groups assessed before (pre) and after (post) the 12-week intervention and the statistical results

Variable	Time point	Training group ($\overline{\boldsymbol{x}}$ ***±***s)	Pre/post (%)	***d***	Control group ($\overline{\boldsymbol{x}}$ ***±***s)	Pre/post (%)	***d***
**Volume (cm**^**3**^**)**							
*m. sternocleidomastoid*	Pre	58.1 ± 12.9	+ 7.4^*^	1.3	46.8 ± 7.4	+ 1.0	0.19
	Post	62.4 ± 12.2			47.4 ± 7.5		
*m. trapezius pars descendens*	Pre	125.4 ± 22.3	+ 8.3^*^	1.1	103.7 ± 26.8	+ 2.0	0.22
	Post	135.8 ± 26.1			105.8 ± 22.3		
Cross-sectional volume of the deep neck muscles (C4)	Pre	60.5 ± 14.5	+ 6.6^*^	0.78	51.3 ± 7.9	+ 2.3	0.27
	Post	64.5 ± 17.8			52.5 ± 8.1		
**Maximum voluntary isometric strength of the cervical spine (Nm)**							
Extension	Pre	56.2 ± 11.3	+ 6.8^*^	0.94	43.0 ± 11.2	−1.0	0.12
	Post	61.1 ± 13.2			42.6 ± 10.2		
Flexion	Pre	34.4 ± 6.8	+ 17.7^*^	1.1	24.9 ± 7.1	+ 10.5^*^	1.1
	Post	40.6 ± 10.2			27.5 ± 7.2		
Lateral flexion to the right	Pre	45.3 ± 10.3	+ 6.9^*^	0.73	32.2 ± 8.0	−1.0	0.52
	Post	48.4 ± 11.4			30.5 ± 6.8		
Lateral flexion to the left	Pre	46.3 ± 11.0	+ 7.3^*^	0.65	33.8 ± 8.5	−1.0	0.18
	Post	49.7 ± 12.4			32.9 ± 8.8		
Rotation to the right	Pre	19.8 ± 6.4	+ 22.7^*^	1.7	12.5 ± 3.6	+ 1.1	0.44
	Post	24.2 ± 7.6			13.6 ± 4.5		
Rotation to the left	Pre	19.3 ± 6.4	+ 23.2^*^	1.5	13.3 ± 3.1	+ 1.1	0.37
	Post	23.8 ± 7.6			14.5 ± 4.5		
**Questionnaire**							
Perception of muscle strain	Pre	2.0 ± 0.45		2.2	2.2 ± 0.75		0.17
	Post	2.4 ± 0.67			2.3 ± 0.52		

^*^ *P* ≤ 0.05 between the pre- and post-test results within each group

The maximum strength for the main cervical spine movements increased significantly from pre-intervention to post-intervention in the training group (spine extension: + 6.8%; flexion: + 17.7%; lateral flexion rightward: + 6.9%; lateral flexion leftward: + 7.3%; rotation rightward: + 22.7%; rotation leftward: + 23.2%). The control group’s maximum strength only increased for spine flexion (spine extension: − 1.0%; spine flexion: + 10.5%; lateral flexion rightward: − 1.0%; lateral flexion leftward: − 1.0%; rotation left and rightward: + 1.1%). The muscle volumes of all examined muscles increased significantly in the training group but not in the control group (training group: *m. sternocleidomastoideus* + 7.4%, *m. trapezius pars descendens* + 8.3%, volume of deep neck muscles + 6.6%; control group: *m. sternocleidomastoideus* + 1.0%, *m. trapezius pars descendens* + 2.0%, volume of deep neck muscles + 2.3%; Table 1).

**Fig. 2:**
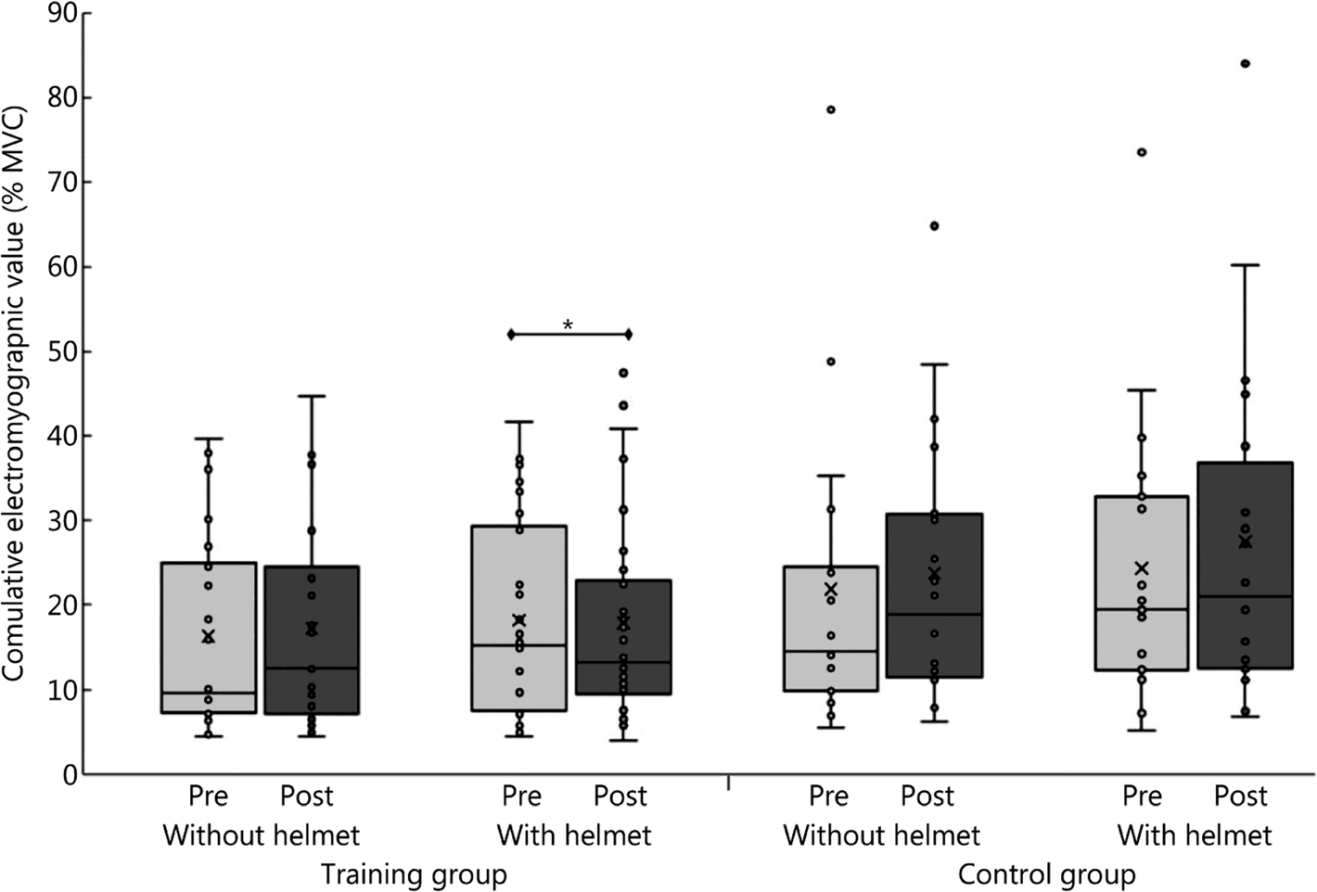
Mean percentage of maximum voluntary contraction of all three muscles studied (i.e., *m. sternocleidomastoideus*, *m. trapezius pars descendens* and *m. erector spinae*) in the training and control groups during all long-arm centrifuge protocols without a helmet and with a helmet before (pre) and after (post) 12 weeks of functional strength training. The “X” in the boxes represents the mean value, and the line represents the median. The box represents the interquartile range (25th–75^th^ percentile). The whiskers demarcate the 5^th^ to the 95^th^ percentile. *P < 0.05

Figure 2 presents the cumulative electromyographic values of both groups during the long-arm centrifuge protocol with and without a helmet before and after the 12-week training program. The %MVC decreased in the training group between the pre-intervention and post-intervention time points when wearing a helmet during the centrifuge runs (*P* = 0.01).

**Fig. 3:**
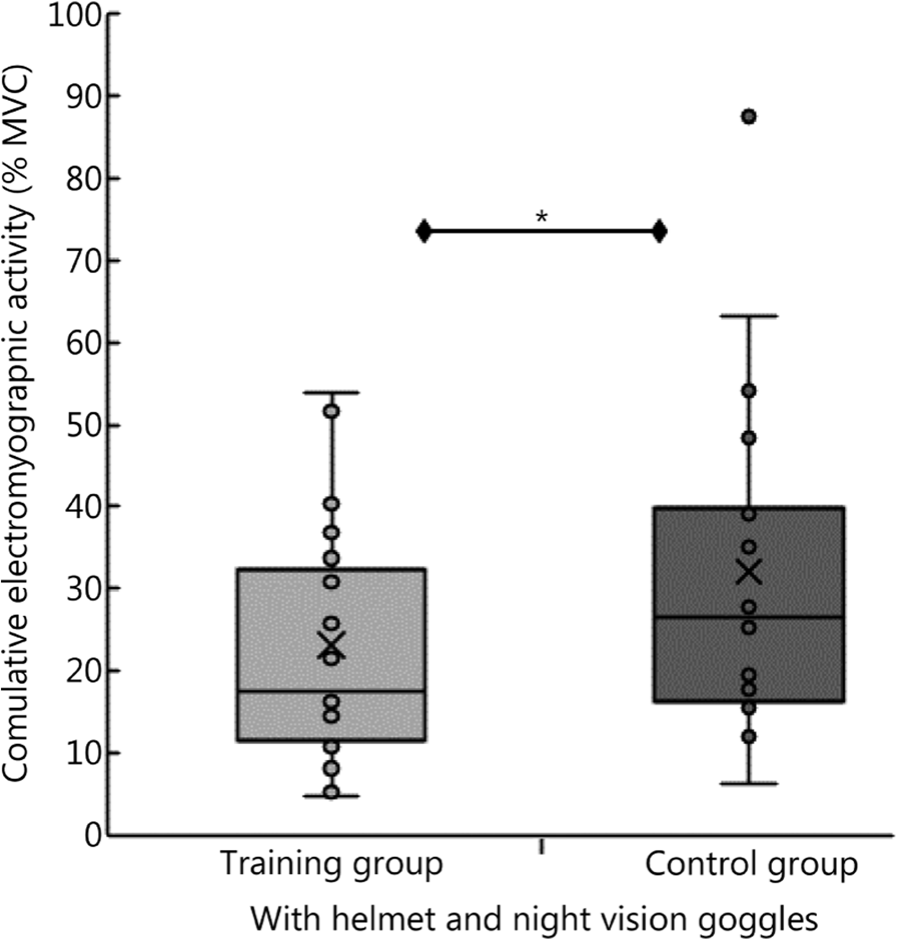
Mean percentages of maximum voluntary contraction of all three muscles studied (i.e., *m. sternocleidomastoideus*, *m. trapezius pars descendens* and *m. erector spinae)* in the training and control groups during all long-arm centrifuge protocols with a helmet and night vision goggles (X-axis). The “X” in each box represents the mean value, and the line represents the median. The box represents the interquartile range (25th–75^th^ percentile). The whiskers demarcate the 5^th^ to the 95^th^ percentile. *P < 0.05

When wearing a helmet and night vision goggles, the control group exhibited higher electromyographic activity than the training group (*P* = 0.003). Figure 3 displays the cumulative electromyographic activity of the two groups when wearing a helmet and night vision goggles.

After the 12-week intervention, there was no significant difference in muscle strain of the neck muscles between the groups or between the pre-intervention and post-intervention time points. However, the increase in effect size from the pre-intervention time point to the post-intervention time point in the training (pre: 2.0 ± 0.45; post: 2.4 ± 0.67; *P* = 0.17; *d* = 0.45) and control (pre: 2.2 ± 0.75; post: 2.3 ± 0.52; *P* = 0.70; *d* = 0.17) groups indicated reduced muscle strain following strength training.

## Discussion

To the best of our knowledge, this is the first study examining the influence of a 12-week functional whole-body strength training program on the volume and strength of the neck and shoulder muscles and muscle activity and strain in high-performance aircraft personnel exposed to flight-specific hypergravity conditions induced by a centrifuge.

The maximum strength and volume of participants’ neck and shoulder muscles increased following 12 weeks of functional strength training. Furthermore, the participants in the training group showed less muscle activation when exposed to elevated G_z_ forces and perceived muscle strain as less stressful than the members of the control group did.

Physiological experiments in high-performance aircraft are challenging, since they require exhaustive approval procedures with unclear outcomes, are expensive, and may require cumbersome measurement devices that are not allowed in the cockpit of a jet for safety reasons. Thus, most experiments seeking to investigate physiological responses in hypergravity are carried out in long-arm centrifuges capable of inducing G-forces in a flight-like setting.

Our initial intention was to incorporate exercises used by participants in sports that also involve acceleration-induced forces to the neck and spine, e.g., bobsledding, motor sports and boxing. Astonishingly, exercise methods for the prevention of acceleration-induced spine injuries in exercise science are scarce and, to the best of our knowledge, are not documented. No accepted form of exercise exists to prevent back and neck pain among jet aviators. Very few studies on special methods for reducing acceleration-induced pain exist; however, a literature review regarding night vision goggle-induced neck pain in military helicopter aircrew suggests that a strong muscular system in the area of the cervical spine has a protective and stabilizing effect when external forces (helmet weight, G_z_ forces or vibration) act on the spine [21].

Additionally, since aircraft personnel are frequently relocated and may have limited access to facilities such as a gym, we sought to develop strength exercises that do not require special strength equipment. Since aircraft personnel are heterogeneous in terms of physical fitness and are exposed to a predominantly sedentary work environment that does not require exercise, we aimed not only to focus on the improvement of neck and shoulder muscle strength but also to incorporate whole-body exercise and a short cardiorespiratory component, as shown in Supplemental Table 1.

The present data show that the maximum strength of the neck muscles increased by 14.1% following 12 weeks of functional strength training. This increase in strength was partly attributable to elevated muscle mass, as supported by the increase in muscle volume, i.e., 6.6% in the deep neck muscles, 7.4% in the sternocleidomastoid muscle and 8.3% in the upper trapezius muscles.

The increases in muscle volume and strength in the present investigation are in line with the findings of other strength studies of similar duration aiming to improve the neck and shoulder muscles in sedentary individuals. Portero et al. [22] quantified neuromuscular cervical adaptations to 8 weeks of strength training in seven healthy men. The MRI-assessed muscle volume increased by 6.4% in the deep neck muscles, 8.8% in the sternocleidomastoid muscles and 12.2% in the upper trapezius muscles, with increases in isometric and isokinetic strength of 35 and 20% [22].

As the main function of deep muscles is to straighten the head and offer stabilization, they have a significant protective influence when external forces, such as elevated G-forces, act on the spine. In the present investigation, participants were not exposed to high accelerations (i.e., + 3 G_z_ compared with inflight measurements, which often exceed + 7 G_z_). We assume that muscle activation would also be lower at + 7 G_z_ following special strength training of the spine muscles. However, we could not expose the participants to higher accelerations in the present study due to the risk of injury and the absence of anti-G protective equipment. Our present data on muscular activity during elevated G_z_ forces are in line with earlier findings investigating the effect of flight helmet mass on cervical erector spinae muscle strain under high G_z_ forces [23, 24].

Hämäläinen [23] reported the results from two test pilots who carried out 16 flights consisting of a series of maneuvers with different helmet masses. The results showed that changing from a heavier to a lighter helmet reduced the mean muscular strain from 9.5 to 8.8% and the MVC from 20.2 to 17.1% at + 4.0 and + 7.0 G_z_. In the present study, the muscle activity of the control group relative to the MVC with a helmet and night vision goggles was significantly higher than that of the training group. Additionally, after the intervention, the muscle strain of the training group was lower than that before the training (with a helmet). The present results indicate that stronger muscles with a greater volume function at a lower relative intensity than smaller muscles during acceleration; thus an increase in muscle volume potentially reduces fatigue during, e.g., longer flights with frequent elevated G_z_ forces. In this regard, Sovelius et al. [25] subjected 16 to two different 6-week training programs (a strength endurance training program and a trampoline training program) and measured the influence on muscle activity during inflight measurements. The authors concluded that both programs reduced the activity of the neck muscles.

We are aware that multiple mechanisms may cause spinal strain complaints. The aim of the present study was not to identify the specific mechanisms of such complaints; for details related to this area, we refer the reader to previous findings [10]. The present aim was to analyze the magnitude of physiological stress on the musculature upon exposure to elevated G_z_ forces and to determine whether the effect of training in this specific environment can be measured. Based on these basic findings, the effects of different training methods (e.g., cardiorespiratory exercise) in reducing strain under G-forces should be compared to determine which program may best prevent spinal injuries.

## Conclusion

Based on the present data, we conclude that a 12-week functional strength training program improves the maximum strength and volume of neck and shoulder muscles. Furthermore, this type of training reduces muscle activation when personnel are exposed to elevated G_z_ forces in a long-arm centrifuge and reduces perceived muscle strain. Further research is warranted to investigate whether stronger shoulder and neck muscles function at a lower relative intensity during acceleration and whether strengthening the neck muscles thus reduces fatigue during longer flights with frequent exposure to elevated G_z_ forces.

## Supplementary Information

**Additional file 1: Supplemental Table 1.** All strength training sessions and their characteristics (repetitions, series, time of recovery, and duration) during the 12-week intervention.

## Data Availability

The datasets used and/or analyzed during the current study are available from the corresponding author upon reasonable request.

## Abbreviations

*d*: Effect size (Cohen’s *d*)
G_z_: Vertical gravity gradient
*m.*: Musculus
MVC: Maximum voluntary contraction
Nm: Newton-meter
