# Supplementary material for: The effects of 12 weeks of functional strength training on muscle strength, volume and activity upon exposure to elevated Gz forces in high-performance aircraft personnel
Source: Mil Med Res. 2021 Feb 23;8:15. doi: 10.1186/s40779-021-00305-8 (PMC7901111; doi:10.1186/s40779-021-00305-8)
Supplement: Supplementary file 1 — Additional file 1: Supplemental Table 1. All strength training sessions and their characteristics (repetitions, series, time of recovery, and duration) during the 12-week intervention. [file 40779_2021_305_MOESM1_ESM.docx]

**Supplemental Table 1** All strength training sessions and their characteristics (repetitions, series, time of recovery, and duration) during the 12-week intervention

| **Week** | **Session** | **Exercise** | **Repetition** | **Series** | **Recovery** | **Duration (min)** |
| --- | --- | --- | --- | --- | --- | --- |
| 1–6 | 1 | **Warm up:**  Rope skipping (5 min)  Shoulder rotation  Front lunges with rotation  Back mobilization  Head rotation  Shoulder rotation (resistance band)  Neck mobilization | 7–10 | 1 |  | 10 |
|  |  | **Strength training:**  Squat presses (resistance band)  Bent over rows (training system with handles)  High pulls (resistance band)  Pushups (medicine ball)  Neck extension (neck trainer)  Mountain climber  Overhead swings (medicine ball)  Neck rotation (additional weight) | 8–12 | 4 | 10 s for changing position and 30 s after each circuit | 40 |
|  |  | **Core stability:**  30-s plank  30-s side plank | 1 | 3 | 10 s for changing position and 30 s after each circuit | 10 |
| 1–6 | 2 | **Warm up:**  Rope skipping (5 min)  Shoulder push ups  Body stretching  Body rotation when sitting  Side lunges  Arm mobility  Shoulder mobility (resistance band) | 7–10 | 1 |  | 10 |
|  |  | **Strength training:**  Face pulls (training system with handles)  Pushups (core wheels)  Front lunges (sandbag)  High pulls (sandbag)  Squat to press (medicine ball)  Upper body rotation (training system with handles)  Neck flexion (neck trainer)  Slams (medicine ball)  Neck rotation (additional weight) | 8–12 | 4 | 10 s for changing position and 30 s after each circuit | 40 |
|  |  | **Core stability:**  30-s side plank  30-s roll out (core wheels) | 1x | 3 | 10 s for changing position and 30 s after each circuit | 10 |
| 1–6 | 3 | **Warm up:**  Rope skipping (5 min)  Reverse butterfly (resistance band)  Shoulder rotation (resistance band)  Squats  Front lunges with body rotation  Head mobility | 7–10x | 1 |  | 10 |
|  |  | **Strength training:**  Reverse butterfly (resistance band)  Shoulder to shoulder press (sandbag)  Front lunges with rotation (medicine ball)  Neck lateral flexion  Deadlifts to pull (resistance band)  Wood choppers (medicine ball)  Back squats (sandbag)  Neck rotation (additional weight) | 8–12 | 4 | 10 s for changing position and 30 s after each circuit | 40 |
|  |  | **Core stability:**  30-s plank  30-s core twist (medicine ball)  30-s hips up (medicine ball) | 1 | 3 | 10 s for changing position and 30 s after each circuit | 10 |
| 6–12 | 1 | **Warm up:**  Rope skipping (5 min)  Head rotation  Neck mobilization to the front  Neck mobilization to the back  Hip mobilization  Side steps  Shoulder mobility (resistance band) | 7-10 | 1 |  | 10 |
|  |  |  |  |  |  |  |
|  |  | **Strength training:**  Back extension rows (training system with handles)  Pushups (medicine ball)  High-pulls (resistance band)  Bent over rows with rotation (resistance band)  Neck extension (neck trainer)  Front lunges rotation (medicine ball)  Neck rotation (additional weight)  Squat to press (medicine ball) | 8-12 | 4 | 10 s for changing position and 30 s after each circuit | 40 |
|  |  | **Core stability:**  30-s core twist (medicine ball)  30-s low plank to high plank | 1 | 3 | 10 s for changing position and 30 s after each circuit | 10 |
| 6–12 | 2 | **Warm up:**  Rope skipping (5 min)  Arm rotation  Head rotation  One leg coordination  Shoulder mobility (resistance band)  Deep squat  Stretch and mobility plank position | 7-10 | 1 |  | 10 |
|  |  | **Strength training:**  Back squats (sandbag)  Deadlifts (training system with handles)  Pushups (resistance band)  Resistance walk (neck trainer)  Overhead swings (medicine ball)  Bent over rows (training system with handles)  Neck rotation (additional weight)  Overhead raises (resistance band) | 8-12 | 4 | 10 s for changing position and 30 s after each circuit | 40 |
|  |  | **Core stability:**  30-s side plank  30-s roll out (core wheels) | 1 | 3 | 10 s for changing position and 30 s after each circuit | 10 |

| 6–12 | 3 | **Warm up:**  Rope skipping (5 min)  Shoulder mobility (resistance band)  Squats  Neck mobilization to the back  Front lunges with rotation | 7-12 | 1 |  | 10 |
| --- | --- | --- | --- | --- | --- | --- |
|  |  | **Strength training:**  Deadlifts to pull (resistance band)  Face pulls (training system with handles)  Pushups with roll outs (core wheels)  Neck extension (neck trainer)  High rows (training system with handles)  Front lunges (sandbag)  Resistance walk (neck trainer)  Squats shoulder to shoulder press (sandbag) | 8-12 | 4 | 10 s for changing position and 30 s after each circuit | 40 |
|  |  | **Core stability:**  30-s sit ups (medicine ball)  30-s swimming-position exercise for back muscles | 1 | 3 | 10 s for changing position and 30 s after each circuit | 10 |
